# The role of filial piety and family dysfunction in eating pathology: a cross-sectional and longitudinal study within the Chinese context

**DOI:** 10.1186/s40337-026-01552-7

**Published:** 2026-03-03

**Authors:** Xu Han, Mei-chun Cheung, Xueni Li, Lei Yang, Chao Chen

**Affiliations:** 1 Department of Social Work, The Chinese University of Hong Kong, Shatin, New Territories, Hong Kong SAR China; 2 Peking University Sixth Hospital, Peking University Institute of Mental Health, NHC Key Laboratory of Mental Health (Peking University), National Clinical Research Center for Mental Disorders (Peking University Sixth Hospital), Beijing, China

**Keywords:** Anorexia, Filial piety, Family functioning

## Abstract

**Background:**

Family functioning encompasses cultural values and behavioral patterns, with family dysfunction referring to pervasive and maladaptive interactions in the latter dimension. In Chinese culture, filial piety is a core familial value, comprising authoritarian filial piety (AFP), rooted in hierarchy and obedience, and reciprocal filial piety (RFP), based on mutual affection and care. This study examines the relationships between AFP, RFP, family dysfunction, and eating pathology among Chinese adults with anorexia nervosa (AN).

**Methods:**

We employed a two-part design that included a cross-sectional analysis of 144 female adults with AN in Chinese mainland and, within the same cohort, a longitudinal follow-up of 75 patients to examine how filial piety and family dysfunction predict changes in eating pathology over time. Correlation, hierarchical multiple regression, and generalized least squares were used for data analysis.

**Results:**

Cross-sectionally, AFP was associated with greater eating pathology (β = 0.161, *p* < .05). While RFP and family dysfunction were not significantly associated with eating pathology after controlling psychological and biological factors. Longitudinally, AFP did not independently predict symptom progression after controlling for baseline pathology, suggesting that AFP may act not as a primary driver of worsening symptoms.

**Conclusion:**

AFP represents a stable cultural risk factor for eating pathology in Chinese adults with AN. These findings underscore the importance of integrating cultural values like AFP into therapeutic frameworks and developing culturally adapted interventions for Chinese populations.

## Plain Language Summary

Family functioning was conceptualized as a construct constituted by two complementary yet distinct dimensions: an abstract dimension of internalized family values and norms, and a concrete behavioral dimension comprising observable processes such as communication, affective involvement, and control. Deficits in the concrete dimension was called family dysfunction. This study investigated the associations between traditional family values—reciprocal filial piety (RFP, grounded in mutual affection and care) and authoritarian filial piety (AFP, based on hierarchical obedience)—and eating pathology among Chinese female adults with anorexia nervosa (AN). Additionally, the role of family dysfunction, was also examined.

To assess the variables of interest, a questionnaire-based approach was employed, incorporating both cross-sectional and longitudinal designs. Data were collected at two time points with a six-month interval between assessments, allowing for the examination of both concurrent relationships and changes over time. The main findings show that AFP was associated with more severe eating pathology. This suggests that AFP acts as a stable cultural background factor in AN. However, family dysfunction and RFP were not associated with eating pathology.

Longitudinally, AFP did not independently predict changes in symptoms over time after accounting for initial severity. The study highlights the importance of considering cultural family values like AFP in understanding and treating AN in Chinese populations, and supports the development of culturally adapted interventions.

## Key Findings

• Authoritarian filial piety (AFP) emerged as a stable and consistent correlate of eating pathology in the cross-sectional dataset, even after controlling psychological and biological factors. However, reciprocal filial piety and family dysfunction are not associated with eating pathology in the cross-sectional dataset.

• Longitudinal analysis indicated that AFP did not independently predict changes in eating pathology over time after controlling the baseline eating pathology. Its influence was primarily concurrent, suggesting it may play a more central role in the initial development or maintenance of eating pathology than in modulating its subsequent trajectory.

• The persistent link between AFP and eating pathology highlights the importance of integrating culturally specific constructs into the etiological models, prevention strategies, and therapeutic approaches for Chinese populations.

## Introduction

Anorexia nervosa (AN) is a severe mental disorder characterized by restricted food intake and significantly low body weight, leading to profound functional impairment, reduced quality of life, and substantial caregiver burden [1]. Although global incidence rates have remained relatively stable, China has experienced a marked rise in the prevalence of AN, with recent estimates exceeding 1.31 million affected individuals [2, 3].

The aetiology and persistence of AN are multifactorial, involving biological, psychological, and interpersonal dimensions. Psychological factors, such as pro-AN beliefs (the valued nature of AN), perfectionism ( as a manifestation of a range of cognitive characteristics), and emotional dysregulation, along with interpersonal factors—particularly family functioning—are strongly implicated in the development and maintenance of the disorder [4]. While some mechanisms may be universal, family functioning is deeply embedded in cultural values and thus vary across societies. Given the increasing prevalence of AN in China [2, 3], there is a critical need to examine culturally specific family functioning factors that may contribute to the development and maintenance of AN.

Family functioning is defined as the dynamic interactions among family members and their collective ability to accomplish essential tasks to meet the biological, psychological, and social goals of the family system [5]. It comprises two primary elements: an abstract element of values and norms, shaped by parents’ internalized experiences, shared family history, and broader cultural influences; and a concrete element encompassing processes such as task accomplishment, role performance, communication, affective involvement, and control [5]. In this study, family dysfunction is conceptualized as an impairment in the concrete element of family functioning.

As part of family functioning, recent studies have increasingly focused on the association between family values and mental symptoms or disorders [6]. Filial piety, a cornerstone of East Asian family value, encapsulates culturally prescribed norms governing children’s attitudes and behaviors toward their parents, encompassing obedience, gratitude, respect, and care [7, 8]. Beyond a mere set of relational rules, it profoundly shapes psychosocial development, influencing individuals’ behavioral patterns even beyond the familial sphere [9]. The dual filial piety model further delineates this construct into two dimensions: reciprocal filial piety (RFP), rooted in affectionate reciprocity cultivated through long-term parent-child interactions, and authoritarian filial piety (AFP), characterized by hierarchical obedience and role-bound obligations [9]. This duality underscores the complex role of filial piety in mental health outcomes [10], as RFP serves a supportive function by reducing the risk of mental disorders, showing a negative association with depression and anxiety in meta-analyses, whereas AFP is positively correlated with externalizing symptoms such as aggression and deviant behaviors, and may also contribute to higher risks of internet addiction and self-harm [10].

Qualitative syntheses also reveal multifaceted manifestations of filial piety in eating pathology. On one hand, filial piety emphasizes bodily integrity and health maintenance as virtuous acts, potentially leading individuals to resist weight loss [11, 12]. On the other hand, eating disorders may evoke guilt due to perceived harm to parents, leading individuals to refrain from seeking assistance from them [13] and serve as indirect expressions of autonomy where direct disagreement is culturally discouraged [12, 14]. Han and Cheung [10] categorized descriptions of filial piety derived from qualitative studies into RFP and AFP, revealing that these two distinct dimensions of filial piety exhibit opposing associations with eating pathology. However, this proposition currently lacks empirical support from quantitative data. Therefore, the current study aims to bridge this gap by empirically examining the role of RFP/AFP in relation to eating pathology among Chinese patients with AN.

## Current study

This study employs a two-part design to examine the relationship between filial piety, family dysfunction and eating pathology among Chinese adults with AN. A cross-sectional investigation aims to assess the concurrent associations among RFP, AFP, family dysfunction, and eating pathology. Building on these findings, a longitudinal investigation seeks to evaluate whether dual filial piety and family dysfunction predicts changes in eating pathology over time.

## Method

### Participants

Participants were recruited from Chinese mainland through staff at specialized hospitals and online platforms. Psychiatrists and psychotherapists reached out to patients who had previously received or were currently undergoing treatment through meetings, personal online platforms, and the instant messaging application WeChat. Researchers also disseminated recruitment information for the study through WeChat public platforms in Chinese mainland that have a significant reach in the promotion of eating disorders. Eligible participants were required to meet the following criteria: (1) a clinician-confirmed diagnosis of AN or atypical AN based on the Diagnostic and Statistical Manual of Mental Disorders, Fifth Edition, Text Revision (1); (2) female; (3) age 18 years or older; and (4) fluency in reading and writing Chinese. Individuals with severe comorbid conditions at the time of the survey (e.g., learning disabilities or psychotic disorders) were excluded.

This study has been approved by the Survey and Behavioral Research Ethics Committee of The Chinese University of Hong Kong. The ethical approval reference number is No. SBRE-23–0250 A. All participants provided informed consent, either online or in written form. Participation was voluntary, and participants were offered a coffee voucher valued at 20 RMB as an incentive.

### Sample size estimation

A priori power analysis was conducted using G^*^Power 3.1 [15] to determine the minimum sample size required for the cross-sectional regression analysis. With nine predictors, an alpha level of 0.05, and a power of 0.80, the analysis indicated a required sample size of 114 participants to detect a small-to-medium effect size. For the longitudinal analysis, a power calculation for the generalized least squares (GLS) model was performed. The analysis indicated that a sample of 126 participants would provide 80.6% power to detect small-to-medium effects (*f²* = 0.15) across two time points with nine predictors, at a significance level of 0.05.

#### Sampling method

A convenience sampling method was used in the current study. This approach was taken because nonprobability samples can be created when the sampling units appear representative and can be conveniently accessed [16, 17]. Consecutive enrollment was also used, meaning that all eligible cases who agree to participate were included in the study. Finally, a total of 144 participants were involved at baseline, of whom 75 also participated at follow-up.

### Variables and measurements

#### Filial piety

The Dual Filial Piety Scale, a Chinese-language scale, uses a 6-point Likert-type rating system to assess each item, ranging from 6 (absolutely essential) to 1 (completely insignificant). Higher scores indicate greater identification with the item, reflecting increased levels of filial piety [9]. The 8-item RFP subscale assesses care, emotional support, and perspective-taking grounded in gratitude (e.g., “Being grateful to my parents for raising me”). Conversely, the 8-item AFP subscale measures deference to parental authority and the subordination of personal needs (e.g., “Giving up one’s aspirations to meet the expectations of parents”). Confirmatory factor analyses in previous studies have consistently validated this two-factor model, supporting its robust construct validity [9]. A significant positive inter-scale correlation was observed between RFP and AFP (*r* = .263, *p* < .01) in this study, confirming that the two constructs are related yet distinct. Both subscales demonstrated good internal consistency across assessment points: the RFP subscale yielded Cronbach’s alpha coefficient of 0.883 at baseline and 0.925 at follow-up, while the AFP subscale showed values of 0.867 at baseline and 0.868 at follow-up.

#### Family dysfunction

The General Functioning Scale of the Family Assessment Device (FAD) was used to evaluate family dysfunction [18]. This scale consists of 12 items and utilizes a 5-point Likert scale ranging from 4 (*strongly agree*) to 0 (*strongly disagree*) [18]. Examples include ‘we feel accepted for what we are,’ ‘we confide in each other,’ and ‘we avoid discussing our fears and concerns.’ (Epstein et al., 1983). The Chinese version demonstrated good reliability and validity through rigorous translation and cultural adaptation assessments [19]. The Cronbach’s alpha coefficient for the scale in this study was 0.909 (baseline) and 0.934 (follow-up).

#### Eating pathology

The Eating Disorder Examination Questionnaire (EDE-Q), designed to assess the range, frequency, and severity of behaviors associated with eating disorders [20, 21] was employed to assess eating pathology. It is categorized into four subscales (Restraint, Eating Concern, Shape Concern and Weight Concern) and has an overall global score, with higher scores indicating more problematic eating difficulties (Fairburn & Beglin, 2008; Mond et al., 2006). The reliability and validity of the Chinese version have been demonstrated through rigorous translation and cultural adaptation assessments [22]. The Cronbach’s alpha coefficient for the scale in this study was 0.949 (baseline) and 0.954 (follow-up).

#### Confounding variables

Body Mass Index (BMI), an indicator of a patient’s physical condition, is considered to potentially influence eating pathology [1] and was therefore included as a confounding variable in the study.

The Depression and Anxiety subscale of the Depression, Anxiety, and Stress Scale (DASS) [23] was used to assess the levels of depression and anxiety among patients. The Chinese version has demonstrated good reliability and validity through rigorous translation and cultural adaptation assessments [24]. The Cronbach’s alpha coefficient for the depression subscale (DASS-D) in this study was 0.919 (baseline) and 0.905 (follow-up), and that for the anxiety subscale (DASS-A) was 0.893 (baseline) and 0.820 (follow-up).

Psychological factors (i.e., pro-AN, perfectionism and emotion dysregulation) associated with the maintenance of eating pathology, are recognized as confounding variables. Pro-AN assessed via the Pros and Cons of Anorexia Nervosa Scale [25, 26], which exhibits a Cronbach’s alpha of 0.930 (baseline) and 0.950 (follow-up) in this study. Additionally, perfectionism is measured using the perfectionism subscale of the Eating Disorder Inventory (EDI) [27, 28], with a Cronbach’s alpha of 0.781 (baseline) and 0.807 (follow-up). Emotional dysregulation is evaluated through the Difficulties in Emotion Regulation Scale (DERS) [29, 30], demonstrating Cronbach’s alpha values of 0.948 (baseline) and 0.953 (follow-up).

### Data analysis

#### Cross-sectional analysis

Cross-sectional analyses were conducted to examine the relationships between family functioning factors, (including RFP & AFP and family dysfunction) and eating pathology. Prior to analysis, normality was assessed; due to non-normal distributions of key variables (such as RFP, AFP, family dysfunction, eating pathology), non-parametric methods were employed. Spearman’s correlations were used to evaluate bivariate associations. Hierarchical regression analyses were performed. Robustness of estimates was enhanced through bootstrapping, given the sample size and distributional characteristics.

#### Longitudinal analysis

Longitudinal analyses utilized data from 75 participants who completed both baseline and follow-up assessments. Attrition analysis was conducted to evaluate potential selection bias. To investigate whether baseline family functioning factors prospectively predicted eating pathology at follow-up, variables from baseline were entered as predictors in a hierarchical regression model predicting eating pathology at follow-up in the paired dataset. Furthermore, generalized least squares (GLS) regression with an autoregressive covariance structure was utilized in the full sample. This methodological approach accounted for the longitudinal data structure and provided robust estimates of the predictive relationships over time.

## Results

### Cross-sectional findings

#### Sample characteristics

A total of 165 questionnaires were distributed, with 144 deemed valid for analysis. Among the valid responses, participants included individuals diagnosed with the AN-restricting subtype (ANR, *n* = 59, 41.0%), the AN-purging subtype (ANP, *n* = 63, 43.8%), and atypical AN (*n* = 22, 15.3%). The proportions of participants who had participated in psychotherapy within the last six months were 49.3% (*n* = 71), while 50.7% (*n* = 73) had not, with no significant difference observed between these groups in terms of eating pathology. Additionally, the analysis revealed no significant differences in eating pathology across the demographic variables examined. Although BMI did not show a significant association within this sample, it was controlled for as a potential biological confounder, given its well-established relationship with eating pathology. Participant sociodemographic characteristics are summarized in Table 1.

Psychological variables (i.e., Pro-AN beliefs, perfectionism, emotion dysregulation, depression, and anxiety) that demonstrated statistically significant associations with eating pathology (as detailed in Table 2) were included as covariates in subsequent analyses.

#### Correlations between dual filial piety, family dysfunction, and eating pathology

Spearman correlation analyses revealed distinct patterns of association among RFP, AFP, family dysfunction, and eating pathology. Higher RFP was significantly associated with lower family dysfunction (*ρ* = -0.336, *p* < .01), while both AFP and family dysfunction were positively correlated with eating pathology (*ρ* = 0.237 and 0.217, respectively, *p* < .01). Table 3 presents the details of spearman correlations among dual filial piety, family dysfunction and eating pathology.

#### Regression analyses between dual filial piety, family dysfunction, and eating pathology

A hierarchical regression analysis was conducted to predict eating pathology. In Model 1 and Model 2, biological (BMI) and psychological factors were entered as controls using forced entry. Model 3 incorporated family functioning factors—family dysfunction and dual filial piety— which explained an additional 3.8% of the variance (*ΔR²* = 0.038, *p* < .05). The final model accounted for 40.5% of the total variance (*R*^*2*^_*adj*_ = 0.365). Among the family functioning factors, only AFP was a significant predictor (*β* = 0.161, *p* < .05). Table 4 presents the detailed results of the hierarchical regression analysis.

### Longitudinal findings

#### Sample characteristics

At the six-month follow-up, 75 participants provided valid data. The sample consisted of 32 (42.7%) with ANR, 32 (42.7%) with ANP, and 11 (14.7%) with atypical AN. Regarding treatment status, 47 (62.7%) did not receive psychotherapy between assessments, while 28 (37.3%) did. Details are presented in Table 5. Among the participants who completed the follow-up assessment, BMI at baseline was significantly associated with eating pathology at follow-up (*ρ =* -0.288, *p* < .05). However, other demographic factors did not show significant differences in follow-up eating pathology.

To evaluate potential attrition bias, demographic variables, psychological factors, family functioning factors and baseline eating pathology were compared between follow-up completers (*n* = 75) and non-completers (*n* = 69). No significant differences were observed in demographic characteristics and family functioning factors (including RFP, AFP and family dysfunction). However, completers reported significantly lower baseline eating pathology, suggesting that participants with less severe symptoms were more likely to be retained in the longitudinal sample. Additionally, completers reported lower emotion dysregulation than non-completers (*p* < .01). Details are presented in Table 6.

#### Longitudinal relationships between dual filial piety, family dysfunction at baseline and eating pathology 6-month follow-up

Spearman correlation analyses on the longitudinal paired sample revealed that family dysfunction (*ρ* = 0.251, *p* < .05) and AFP (*ρ* = 0.245, *p* < .05) at baseline were significantly correlated with eating pathology at follow-up. No significant correlations were observed between RFP at baseline and eating pathology at follow-up. Details are presented in Table 7.

#### Longitudinal associations of dual filial piety and family dysfunction at baseline with eating pathology at 6-month follow-up

A hierarchical regression analysis was conducted to examine whether family functioning factors predicted eating pathology at follow-up. Baseline eating pathology was entered in the first step, explaining a substantial proportion of variance in follow-up symptoms (*R²* = 0.712, *F*_(1, 73)_ = 180.07, *p* < .001). In successive models (Models 2, 3, and 4), we incrementally added sets of covariates representing biological, psychological, and family functioning factors, respectively. In the final model, which included all predictors, baseline eating pathology remained the strongest and only consistently significant predictor (*β* = 0.818, *p* < .001). While perfectionism showed a significant positive association (*β* = 0.146, *p* = .044), none of the family functioning factors—family dysfunction, RFP, and AFP—significantly predicted follow-up pathology after controlling for baseline symptoms and other covariates (all *p*s > 0.05). The inclusion of these family functioning factors in the final step did not meaningfully improve the model fit, as indicated by a minimal increase in *R²* (*ΔR²* = 0.009). Detailed results are presented in Table 8.

As a supplementary analysis, we tested an alternative hierarchical multiple regression model in which baseline eating pathology was entered in the final step. Biological and psychological factors were entered as a block in Models 1 and 2, respectively, followed by the stepwise entry of family functioning factors in Model 3. AFP significantly predicted follow-up pathology (*β* = 0.220, *p* = .034, *ΔR²* = 0.042). However, when baseline pathology was added in the final step, it became the strongest predictor (*β* = 0.816, *p* < .001, *ΔR²* = 0.369), and the association between AFP and follow-up pathology was no longer significant. This indicates that the predictive effect of AFP may not be independent of pre-existing symptom levels. Details are presented in Table 9.

#### Dual filial piety and family dysfunction and their interactions with time as predictors of eating pathology

A longitudinal GLS model was fitted to examine changes in eating pathology over two time points. The model included AFP, RFP, and family dysfunction as predictors, along with their interactions with time. BMI and psychological variables—pro-AN, perfectionism, emotion dysregulation, depression and anxiety—were mean-centered and included as covariates.

The autoregressive AR [1] covariance structure yielded a rho of 0.679 (*p* < .001), indicating moderate positive autocorrelation, which suggests that individual eating pathology scores exhibited considerable stability over time between the two waves. Among family functioning factors, only AFP approached marginal significance (*p* = .075), while RFP and family dysfunction were non-significant. None of the interaction terms with time reached significance, indicating that the relationships between these familial variables and eating pathology remained stable across time. Details are presented in Table 10.

## Discussion

Filial piety is a fundamental value that influences the attitudes of children towards their parents in Chinese culture and other East Asian communities [31]. This study aimed to explore the relationship between dual filial piety and eating pathology among Chinese patients with AN to address a gap in the literature regarding the association between dual filial piety and this mental health condition.

Based on the dual filial piety model, RFP emphasizes a bidirectional emotional connection and responsibility, where the relationship between children and parents is mutual [9]. RFP focuses on emotional exchange and mutual support, with children expressing gratitude and care for their parents while also expecting understanding and support in return from them [9]. The present study, however, found no significant association between RFP and eating pathology within a clinical AN sample. This contrasts with its established protective role against general internalizing symptoms like depression and anxiety, as evidenced in community-based meta-analyses [10]. This discrepancy may be attributed to the restricted range of eating pathology severity inherent in a clinical sample, which statistically reduces the likelihood of detecting significant associations. More importantly, it underscores the domain-specificity of protective psychological mechanisms—what buffers against general distress in the community may not directly translate to buffering core symptoms within a specific, severe disorder. RFP’s emotional resources may buffer diffuse psychological distress but appear insufficient to counteract the specific cognitive-behavioral core of eating pathology. Furthermore, in the clinical context of AN, powerful maintaining factors (e.g., cognitive distortions, low BMI) likely dominate the psychopathological landscape [32], diminishing the observable influence of broader familial assets. The correlation between RFP and AFP also suggests that in clinical families, the risk-enhancing context of high AFP may obscure or suppress the protective potential of RFP, indicating a complex interaction that warrants further study.

AFP emphasizes self-sacrifice, obedience and respect towards parents, typically associated with traditional authority relationships [9]. It embodies a unidirectional sense of responsibility, wherein children are expected to unconditionally support and comply with the expectations and demands of their parents [9]. The significant positive correlations between AFP and eating pathology suggests that notions related to family hierarchy, obedience, and self-sacrifice [33, 34] may be associated with higher eating pathology. Notwithstanding the limitations in sample size and statistical power which preclude definitive mechanistic conclusions, several non-mutually exclusive hypotheses may be proposed to interpret the observed association between AFP and eating pathology. First, AFP is closely tied to self-sacrifice [9], which may promote individual needs suppression and use eating pathology as a maladaptive means of self-gratification and assertion as a common factor observed across various cultural contexts, such as in Israel [35]. Second, AFP correlates strongly with traditional values and lower modernity [9], suggesting that individuals high in AFP may experience heightened cultural conflict, potentially manifesting as a “culture-change syndrome” such as AN [36]. Furthermore, AFP reflects aspects of indigenous Chinese personality, including relational orientation (renqing) and saving faces (mianzi), which emphasize the pursuit of harmony [9]. These cultural values may contribute to conflict avoidance, the utilization of eating pathology as an outlet, and stigma, thereby acting as barriers to help-seeking behaviors. Lastly, AFP has been empirically linked to certain personality traits, such as higher neuroticism and lower extraversion [9], which are established risk factors for eating disorders [37]. However, this pathway remains partially unexplained, as RFP, which demonstrates stronger correlations with such personality dimensions in the study of Yeh and Bedford [9], was not significantly associated with pathology in our data.

Although family dysfunction was significantly correlated with eating pathology in bivariate analysis, its independent predictive effect became non-significant in a multiple regression that included psychological factors and AFP. The results suggest that the broad influence of family dysfunction may be mediated through more specific cognitive-ethical frameworks.

For AFP and family dysfunction, while longitudinal data indicate a correlation with subsequent eating pathology, these factors did not remain significant predictors after controlling for baseline symptoms. This suggests that their influence is likely mediated through pre‑existing levels of pathology rather than directly driving symptom change over time. The non‑significant interaction between family functioning factors and time further implies that their association with eating pathology remains stable across the study period—neither strengthening nor weakening—which supports their characterization as relatively stable contextual vulnerability factors. Therefore, family functioning may appear to co‑occur with eating pathology, forming a concurrent psychosocial symptom cluster. These elements are interrelated and mutually reinforcing, likely sharing common environmental or biological vulnerabilities, however they do not necessarily constitute a clear antecedent‑consequence sequence. This finding is consistent with previous research on the relationship between family functioning and eating pathology [38].

## Clinical implications

The motivation for this study originates from recurrent clinical observations of eating pathology disrupting family harmony in Chinese households. Within these families, filial piety is often emphasized as a resource for mobilizing responses to familial crises. We have observed that some patients who exhibit compliant behaviors may experience short-term improvements in eating symptoms; however, others appear to face escalated family conflict or crisis.

The clinical implications of this study are multifaceted. First, from an individual perspective, a nuanced understanding of filial piety is essential. Traditional interpretations that prioritize obedience, hierarchy, and self-sacrifice [39] may not support personal development, psychological well-being, or recovery. Encouraging flexible and reciprocal expressions of filial piety—rather than rigid adherence to obedience and self-sacrifice—may represent a more adaptive strategy for supporting recovery from eating pathology.

Second, from a family systems perspective, it is critical to move beyond a strict reliance on AFP to maintain family order, especially in multigenerational households. For parents or caregivers, this implies avoiding the use of filial appeals to secure compliance around eating during episodes of illness. Instead, clear, direct communication of expectations and the cultivation of a revised, supportive authority are recommended.

Third, at a broader conceptual level, it is important to note that eating pathology demonstrates notably weak associations with general measures of family dysfunction. This distinction underscores that existing interventions targeting family dysfunction—while valuable—are insufficient to address the specific cultural and relational dynamics embodied in filial piety. Consequently, systematic understanding and intervention focused explicitly on filial piety remain irreplaceable in clinical contexts where eating pathology and family harmony intersect.

Fourth, at a societal level, AN itself can be understood as a manifestation of a “cultural change syndrome.” [40] This conceptualization is particularly relevant when considering how traditional cultural scripts, such as rigid formulations of filial piety, clash with modern individualistic values. This conflict can transform culturally sanctioned values into sources of pathological expression. Therefore, in clinical practice and for broader social harmony, it is vital to promote a more flexible, contemporary understanding of such traditions, fostering their constructive evolution.

## Limitations

This study has several limitations. First, the relatively small sample size, due to the low population prevalence of AN [2], may have constrained effect sizes. This limitation is crucial for contextualizing the non-significant findings for family functioning factors and eating pathology, as it raises the possibility that the lack of significance may be attributable to insufficient power rather than a true absence of effect. The relatively small sample size also limited the depth of mechanistic exploration between filial piety and eating pathology.

Second, the longitudinal dataset was subject to significant attrition, particularly among severe cases at baseline, resulting in a retained sample that may predominantly represent a less severe clinical subgroup. This attrition introduces the potential for selection bias and may limit the generalizability of the findings. Consequently, the observed association between family functioning factors and eating pathology should be interpreted with caution, as its magnitude and applicability may be attenuated. An untestable but plausible assumption is that the role of family functioning factors like AFP appears attenuated because excluding participants with more severe eating pathology in the longitudinal dataset restricted the range of this variable. This range restriction would reduce the statistical power to detect a significant association, potentially leading to an underestimation of the true relationship.

Third, the study was limited to a clinical AN sample, which restricted the range of eating pathology severity and likely reduced statistical power to detect associations with family functioning factors.

Finally, the limited time intervals between follow-up assessments may have constrained the exploration of longer-term relational dynamics. Given that both filial piety and eating pathology tend to change gradually, this temporal restriction could have impacted our ability to capture these dynamics more comprehensively.

## Future direction

This study highlights several directions for future research. The hypothesized mechanisms linking AFP to eating pathology remain theoretical and warrant empirical testing the potential mediators such as personality traits, self-sacrifice, and cultural value negotiation. Besides that, future studies should include community samples to examine these relationships across a broader spectrum of eating pathology severity. Furthermore, this work underscores the importance of incorporating indigenous psychological constructs, such as filial piety, into etiological models of psychopathology, which may diverge from Western-centric frameworks and inform culturally responsive interventions.

**Table 1:** Sociodemographic and Clinical Characteristics of Chinese Patients with Anorexia Nervosa (*N* = 144)

Variable	Option	*n* (%)/median (IQR)
Age (year)	-	24 (22–28)
Education	Middle school	8 (5.6%)
	Undergraduate and above	134 (93.1%)
Occupation	At work	44 (30.6%)
	In school	73 (50.7%)
	Other and not engaged	24 (16.7%)
BMI (kg/m^2^)	-	18.35 (16.50-20.76)
Lowest BMI from the Onset (kg/m^2^)	-	14.06 (12.82–16.50)
Diagnosis	ANP	63 (43.8%)
	ANR	59 (41.0%)
	Atypical AN	22 (15.3%)
Illness Duration (months)	-	72 (48–100)
Treatment Status	Not receiving psychotherapy at the last six months	73 (50.7%)
	Receiving psychotherapy at the last six months	71 (49.3%)

IQR: interquartile range; BMI: body mass index; ANP: anorexia nervosa, purging type; ANR: anorexia nervosa, restricting type

**Table 2:** Descriptive Statistics and Bivariate Correlations of Study Variables with Eating Pathology

Variable	Median (IQR)	Spearman’s ρ with Eating Pathology	*p*
RFP	40.00 (35.00–44.00)	0.009^a^	0.917
AFP	18.00 (13.00–23.00)	**0.237** ^**a**^	**0.004**
Family Dysfunction	28.00 (22.00–34.00)	**0.217** ^**a**^	**0.009**
Pro-AN	-0.66 (-1.19 - -0.13)	**0.384** ^**a**^	**< 0.001**
Perfectionism	28.50 (24.00–31.00)	**0.165** ^**a**^	**0.048**
Emotion Dysregulation	56.00 (40.00–64.00)	**0.416** ^**a**^	**< 0.001**
Depression	9.00 (5.00–14.00)	**0.455** ^**a**^	**< 0.001**
Anxiety	8.00 (4.00-12.75)	**0.347** ^**a**^	**< 0.001**

Bold indicates statistical significance (*p* < .05)

IQR: interquartile range; RFP: reciprocal filial piety; AFP: authoritarian filial piety

**Table 3:** Spearman Correlation Coefficients among Family Functioning Factors and Eating Pathology for Baseline

Variable 1	Variable 2	ρ
Family Dysfunction	RFP	**− 0 0.336** ^**^
Family Dysfunction	AFP	− 0 0.081
Family Dysfunction	Eating Pathology	**0.217** ^**^
RFP	AFP	**0.263** ^**^
RFP	Eating Pathology	0.009
AFP	Eating Pathology	**0.237** ^**^

Bold indicates statistical significance (^*^p < .05, ^**^p < .01)

RFP: reciprocal filial piety; AFP: authoritarian filial piety

**Table 4:** Hierarchical Regression Analysis Predicting Eating Pathology at Baseline

Independent variables	Model 1	Model 2	Model 3
	B(β) [95% BootCI]	B(β) [95% BootCI]	B(β) [95% BootCI]
Constant	2.907^***^	2.907^***^	2.907^***^
	[2.640, 3.160]	[2.666, 3.114]	[2.667, 3.107]
Biological factors			
BMI	– 0.039 (– 0.094)	– 0.003 (– 0.006)	0.015 (0.037)
	[– 0.106, 0.026]	[– 0.060, 0.052]	[– 0.051, 0.069]
Psychological factors			
Pro-AN	–	0.646^***^ (0.291)	0.630^***^ (0.284)
		[0.318, 0.957]	[0.310, 0.938]
Perfectionism	–	– 0.014 (– 0.050)	– 0.029 (– 0.102)
		[– 0.052, 0.032]	[– 0.071, 0.022]
Emotion dysregulation	–	0.031^***^ (0.297)	0.032^***^ (0.303)
		[0.013, 0.048]	[0.014, 0.048]
Depression	–	0.069^*^ (0.265)	0.057^*^ (0.219)
		[0.013, 0.124]	[0.001, 0.116]
Anxiety	–	0.001 (0.003)	– 0.002 (– 0.006)
		[– 0.058, 0.058]	[– 0.059, 0.056]
Family functioning factors			
Family dysfunction	–	–	0.027 (0.124)
			[– 0.011, 0.063]
RFP	–	–	0.019 (0.079)
			[– 0.021, 0.058]
AFP	–	–	0.036^*^ (0.161)
			[– 0.0002, 0.071]

A dash (“−”) indicates that the variable was not included in the regression model

RFP: reciprocal filial piety; AFP: authoritarian filial piety

^*^p < .05

^**^p < .01

^***^p < .001

**Table 5:** Sample Characteristic in the Longitudinal Dataset (*n* = 75)

Variable	Option	*n* (%)/Median (IQR)
Demographic characteristics		
Age at baseline (year)	–	24 (22–28)
Education	Middle school	4 (5.3%)
	Undergraduate and above	71 (94.7%)
Occupation	Employed	23 (30.7%)
	Student	40 (53.3%)
	Other and not engaged	11 (14.7%)
Clinical characteristics		
BMI at baseline (kg/m^2^)	–	18.62 (17.02–21.05)
BMI at follow-up (kg/m^2^)	–	19.23 (17.15–21.43)
Lowest BMI from the onset (kg/m^2^)	–	14.15 (12.87–16.60)
Diagnosis	AN-P	32 (42.7%)
	AN-R	32 (42.7%)
	Atypical AN	11 (14.7%)
Illness duration at baseline (month)	–	75 (48–108)
Treatment status at baseline	Not receiving psychotherapy	37 (49.3%)
	Receiving psychotherapy	38 (50.7%)

IQR: interquartile range; BMI: body mass index; ANP: anorexia nervosa, purging type; ANR: anorexia nervosa, restricting type

**Table 6:** Group comparison between follow-up and non-follow-up participants in the longitudinal dataset

Variable	Option	Follow-up Group (*n* = 75)		Non-Follow-up Group (*n* = 69)		U/χ²	*p*
		Median/Frequency	IQR/Percent	Median/Frequency	IQR/Percent		
Demographic characteristics							
Age (years)	-	24	22–28	24	22-27.50	2514.0^a^	0.768
Education	Middle school	4	5.30%	4	5.80%	0.027^b^	0.869
	Undergraduate and above	71	94.70%	63	91.30%		
Occupation	At work	23	30.70%	21	30.40%	0.583^b^	0.747
	In school	40	53.30%	33	47.80%		
	Other and not engaged	11	14.70%	13	18.80%		
Clinical characteristics							
BMI (kg/m^2^)	-	18.63	17.02–21.04	18.14	15.90–20.20	2122.5^a^	0.063
Lowest BMI from the Onset (kg/m^2^)	-	14.15	12.87–16.60	14	12.55–16.50	2300.5^a^	0.545
Diagnosis	ANP	32	42.70%	31	44.90%	0.190^b^	0.909
	ANR	32	42.70%	27	39.10%		
	Atypical AN	11	14.70%	11	15.90%		
Illness Duration (months)	-	75	48–108	72	48–96	2279.0^a^	0.487
Psychological Interventions	Not receiving psychotherapy	37	49.30%	36	52.20%	0.116^b^	0.733
	Receiving psychotherapy	38	50.70%	33	47.80%		
Eating Pathology	-	2.33	1.75–3.71	3.75	2.35–4.70	**1736.0** ^**a**^	**0.001**
Study variables							
RFP	-	39	33.00–44.00	40	36.00-44.50	2388.0^**a**^	0.424
AFP	-	17	13.00–23.00	18	12.50–23.00	2546.0^**a**^	0.868
Family Dysfunction	-	28	22.00–34.00	29	23.00–34.00	2445.5^**a**^	0.570
Pro-AN	-	-0.69	-1.36–-0.20	-0.59	-1.08–-0.14	2257.0^**a**^	0.186
Perfectionism	-	28	24.00–31.00	29	23.00–33.00	2409.0^**a**^	0.474
Emotion Dysregulation	-	51	38.00–60.00	59	48.50–68.50	**1903.0** ^**a**^	**0.006**
Depression	–	10	5–13	9	5–15	2547.5^a^	0.873
Anxiety	–	9	4–13	8	3.5–12.5	2749.5^a^	0.516

Bold indicates statistical significance (*p* < .05). a Mann-Whitney U. b Chi-Square Statistic

IQR: interquartile range; BMI: body mass index; ANP: anorexia nervosa, purging type; ANR: anorexia nervosa, restricting type; RFP: reciprocal filial piety; AFP: authoritarian filial piety

**Table 7:** Spearman’s Correlations among Family Functioning Factors and Eating Pathology at Baseline and Follow-up in the Longitudinal Dataset

Variable	1	2	3	4	5	6	7	8
1. Family Dysfunction (Baseline)	–							
2. RFP (Baseline)	− 0.336^**^	–						
3. AFP (Baseline)	− 0.081	0.263^**^	–					
4. Eating Pathology (Baseline)	0.217^**^	0.009	0.237^**^	–				
5. Family Dysfunction (Follow-up)	0.695^**^	− 0.286^*^	− 0.034	0.184	–			
6. RFP (Follow-up)	− 0.360^**^	0.713^**^	0.326^**^	0.061	− 0.289^*^	–		
7. AFP (Follow-up)	− 0.149	0.340^**^	0.698^**^	0.273^*^	0.005	0.351^**^	–	
8. Eating Pathology (Follow-up)	0.251^*^	0.076	0.245^*^	0.827^**^	0.203	0.097	0.196	–

RFP: reciprocal filial piety; AFP: authoritarian filial piety

^*^p < .05

^**^p < .01

**Table 8:** Regression Coefficients Predicting Follow-up Eating Pathology in the Longitudinal Dataset with Baseline Pathology Entered First

Predictor	Model (baseline adjusted)			
	B	SE	β	*p*
(Constant)	2.68	0.097		< 0.001
Eating pathology (baseline)	0.802	0.079	0.818	< 0.001
BMI	-0.021	0.024	-0.057	0.391
Pro-AN	-0.111	0.148	-0.052	0.457
Perfectionism	0.04	0.02	0.146	0.044
Emotion dysregulation	0.011	0.008	0.107	0.161
Depression	-0.002	0.025	-0.008	0.937
Anxiety	-0.009	0.024	-0.034	0.715
Family dysfunction	0.014	0.015	0.07	0.343
RFP	0.025	0.016	0.117	0.118
AFP	-0.008	0.015	-0.039	0.584
Model Statistics	R² = 0.773		Radj² = 0.738	F = 21.81

BMI: body mass index; RFP: reciprocal filial piety; AFP: authoritarian filial piety

^*^p < .05

^**^p < .01.

^***^p < .001

**Table 9:** Regression coefficients predicting follow-up eating pathology in the longitudinal dataset with baseline pathology entered last

Predictor	Model 3 (prospective)				Model 4 (baseline adjusted)			
	B	SE	β	*p*	B	SE	β	*p*
(Constant)	2.401	0.148		< 0.001	2.662	0.097		< 0.001
Biological factor								
BMI	-0.003	0.037	-0.007	0.944	-0.027	0.023	-0.075	0.251
Psychological factors								
Pro-AN	0.097	0.223	0.045	0.664	-0.174	0.143	-0.081	0.226
Perfectionism	-0.002	0.03	-0.007	0.951	0.045	0.019	0.164	0.021
Emotion dysregulation	0.036	0.011	0.357	0.002	0.01	0.008	0.099	0.197
Depression	0.069	0.037	0.286	0.064	0.003	0.024	0.01	0.916
Anxiety	-0.006	0.039	-0.021	0.884	-0.007	0.024	-0.028	0.76
Family functioning factor								
AFP	0.045	0.021	0.22	0.034	-0.002	0.014	-0.008	0.912
Baseline pathology								
Eating pathology (W1)	–	–	–	–	0.8	0.079	0.816	< 0.001
Model Statistics	R² = 0.395	R_adj_ ² = 0.332	F = 6.12^***^		R² = 0.764	R_adj_² = 0.749	F = 58.42^***^	
	DW = 1.924							

BMI: body mass index; RFP: reciprocal filial piety; AFP: authoritarian filial piety

^*^p < .05

^**^p < .01

^***^p < .001

**Table 10:** Analysis of Dual Filial Piety, Family Dysfunction and Their Interactions with Time as Predictors of Eating Pathology in the Longitudinal Dataset

Type	Parameter	Estimate	SE	df	t	*p*	95% Confidence interval	
							Lower bound	Upper bound
	Intercept	2.623	0.895	203.000	2.932	0.004	0.859	4.387
Biological factors	BMI	0.002	0.026	175.397	0.083	0.934	-0.049	0.053
Psychological factors	Pro-AN	0.549	0.130	201.995	4.223	0.000	0.293	0.806
	Perfectionism	-0.018	0.017	202.791	-1.016	0.311	-0.052	0.017
	Emotion dysregulation	0.027	0.007	198.595	4.087	0.000	0.014	0.040
	Anxiety	0.003	0.023	191.071	0.153	0.878	-0.041	0.048
	Depression	0.046	0.021	184.985	2.170	0.031	0.004	0.087
Family functioning factors	Family dysfunction	0.016	0.015	198.242	1.063	0.289	-0.013	0.045
	RFP	0.013	0.016	193.640	0.838	0.403	-0.018	0.044
	AFP	0.026	0.015	201.912	1.789	0.075	-0.003	0.055
Interactions	Family dysfunction ^*^ time	-0.011	0.018	89.361	-0.628	0.532	-0.048	0.025
	RFP ^*^ time	0.016	0.022	84.279	0.733	0.465	-0.027	0.059
	AFP ^*^ time	-0.028	0.020	76.157	-1.415	0.161	-0.067	0.011
Time	[time = 0]	0.195	0.130	87.320	1.506	0.136	-0.062	0.453

The time variable was coded as 0 for baseline and 1 for follow-up. The interaction terms (e.g., AFP × time) test whether the effect of the predictor on the outcome changed between assessment waves. [time = 0] corresponds to the baseline measurement

BMI: body mass index; RFP: reciprocal filial piety; AFP: authoritarian filial piety

^*^p < .05

^**^p < .01

## Data Availability

The data that support the findings of this study are available from the corresponding author upon reasonable request.

## Abbreviations

AN: Anorexia nervosa
ED: Eating disorder
RFP: Reciprocal filial piety
AFP: authoritarian filial piety
